# Biocompatible Electrospun Polycaprolactone-Polyaniline Scaffold Treated with Atmospheric Plasma to Improve Hydrophilicity

**DOI:** 10.3390/bioengineering8020024

**Published:** 2021-02-13

**Authors:** Michela Licciardello, Gianluca Ciardelli, Chiara Tonda-Turo

**Affiliations:** 1 Department of Mechanical and Aerospace Engineering, Politecnico di Torino, 10129 Turin, Italy; michela.licciardello@polito.it (M.L.); gianluca.ciardelli@polito.it (G.C.); 2 Polito BIOMedLAB, Politecnico di Torino, 10129 Turin, Italy; 3 Interuniversity Center for the Promotion of the 3Rs Principles in Teaching and Research, 56122 Pisa, Italy; 4 CNR-IPCF, National Research Council-Institute for Chemical and Physical Processes, 56124 Pisa, Italy

**Keywords:** tissue engineering, conductive materials, electrospinning, cold atmospheric plasma treatment

## Abstract

Conductive polymers (CPs) have recently been applied in the development of scaffolds for tissue engineering applications in attempt to induce additional cues able to enhance tissue growth. Polyaniline (PANI) is one of the most widely studied CPs, but it requires to be blended with other polymers in order to be processed through conventional technologies. Here, we propose the fabrication of nanofibers based on a polycaprolactone (PCL)-PANI blend obtained using electrospinning technology. An extracellular matrix-like fibrous substrate was obtained showing a good stability in the physiological environment (37 °C in PBS solution up 7 days). However, since the high hydrophobicity of the PCL-PANI mats (133.5 ± 2.2°) could negatively affect the biological response, a treatment with atmospheric plasma was applied on the nanofibrous mats, obtaining a hydrophilic surface (67.1 ± 2°). In vitro tests were performed to confirm the viability and the physiological-like morphology of human foreskin fibroblast (HFF-1) cells cultured on the plasma treated PCL-PANI nanofibrous scaffolds.

## 1. Introduction

Tissue engineering (TE) is an innovative strategy that aims to repair and/or restore damaged tissues by combining biomaterial scaffolds, cells and additional bioactive cues into functional tissues [1]. The main goal of TE approaches is the design of scaffolds that are suitable for recreating the physiological microenvironment for cell growth [2]. The topographical, physical and biochemical properties of these structures can be tailored to trigger specific signals, which regulate cell attachment, viability, proliferation and differentiation [3].

In recent decades, the attention of the scientific community has been focused on the potentiality—within this framework—of conductive polymers (CPs); an interesting class of ionic electroactive organic polymers that combine the optical and electrical properties of both metallic and inorganic semiconducting materials with the versatility and processability of synthetic polymers [4,5]. Their conductive behavior is the result of the presence of conjugated double bonds in their molecular structure [6]. In their neutral basic form, the conductivity of CPs is very low and ranges from 10^−10^ to 10^−5^ S cm^−1^. However, it can be raised by using the chemical/electrochemical redox doping process that enhances the electric charge mobility within the polymeric chains [7]. The exploitation of these materials for biomedical applications is mainly a consequence of their attractive physical, chemical and electrical properties. Several CPs, such as polypyrrole (PPy), polyaniline (PANI), poly(3,4-ethylenedioxythiophene) (PEDOT), are currently employed as biomaterials for TE approaches, thanks to their ability to modulate the functions of different cell phenotypes [8]. CP-based scaffolds have been reported to promote nerve [9,10,11], bone [12,13] and cardiac [14,15] cell growth and differentiation with good cell viability, high expression levels of tissue specific proteins and an increase in neurite outgrowth being observed. Likewise, the use of CPs has largely been investigated in the context of healing and regenerating wounds [16,17], as well as for vascular tissue engineering applications [18,19]. Polyaniline (PANI) is one of the most widely studied CP types in TE applications, thanks to its easy synthesis, good conductivity and thermical/chemical stability [20,21]. Depending on the polymer oxidation level, three different forms of PANI can be identified: (i) leucoemeraldine (fully reduced form); (ii) pernigraniline (fully oxidized form); emeraldine base (semi-oxidized form) [20,22]. Among these, the emeraldine base is the most frequently used because it is the only one that is conductive and stable in air [20,22]. Its conductive form, PANI emeraldine salt, is obtained by doping the PANI emeraldine base with protonic acids [23,24]. Nevertheless, the use of PANI for TE scaffold is limited by its weak processability, mechanical behavior and biodegradability [20]. To overcome these limitations, blending with conventional biodegradable polymers is often required to permit processability through conventional manufacturing processes [5].

In the TE field, a large number of techniques are employed in the fabrication of polymeric scaffolds that are capable of reproducing the specific behavior of the extracellular matrix (ECM) in native tissues [25,26,27,28,29,30,31], which is characterized by a fibrous architecture. Fibrous polymeric scaffolds are commonly produced through the simple and versatile process of electrospinning, that allows one to sequentially fabricate micro- or nano-fibers by mimicking the structure of the natural ECM [32]. Recently, many experimental studies have reported the successful production of electrically active electrospun membranes by blending PANI with biodegradable and flexible poly(*ε*-caprolactone) (PCL) for culturing several electrically excitable cell lines. Chen et al. [33] produced aligned conductive PCL-PANI electrospun nanofibers for skeletal muscle TE by culturing C2C12 myofibroblasts. They demonstrated that the combined effect of scaffold topography and conductivity cues resulted in increases in C2C12 differentiation. Furthermore, a PCL-PANI high conductive scaffold was obtained by electrospinning the solution with a high concentration of PANI [34]. The obtained three-dimensional structure supported the infiltration and proliferation of bone marrow-derived mesenchymal stem cells (MSCs). Garrudo et al. [35] investigated the attachment, viability and growth of neural stem cells (NSCs) seeded on PCL-PANI electrospun membranes for neural TE. Moreover, Hanumantharao et al. [36] recently assessed the use of a PCL-PANI honeycomb electrospun scaffold for wound healing applications, by culturing adult human dermal fibroblast (HDFa) cells on the scaffold.

In this work, biocompatible and biodegradable PCL was selected as the core material to facilitate the processability of PANI into nanofibrous mats. Electrospinning parameters were optimized to obtain nanofibrous and defect-free membranes, then, a complete investigation of the chemical–physical and mechanical properties of the optimized electrospun mats was performed. PCL-PANI mats showed a high level of hydrophobicity which can strongly reduce cell adhesion, hindering cell growth [37]. To overcome this limitation, the treatment of PCL-PANI electrospun mats with cold atmospheric argon (Ar) plasma was proposed for the first time. Cold atmospheric Ar plasma was investigated for its ability to impart hydrophilic properties to the surface of PCL-PANI fibers, minimizing the poor cell adhesion ascribed to the hydrophobic behavior of PCL-PANI scaffolds that has been reported in the literature so far [14,38]. Cold atmospheric argon (Ar) plasma is a non-thermal process that permits the functionalization of high temperature-sensitive materials, without compromising their physical–chemical properties and morphology. Indeed, cold atmospheric Ar plasma consists of low-temperature particles [39] as the gas is generated at room temperature and atmospheric pressure in an open-air configuration [40,41]. Plasma treatment was performed on PCL-PANI electropsun mats showing reductions in contact angle values, without affecting membrane morphology. Then, the attachment, viability and proliferation of HFF-1 human fibroblasts on the PCL-PANI nanofibrous mats, prior to, and after plasma treatment, were evaluated to explore the use of these substrates in TE applications.

## 2. Materials and Methods

### 2.1. Solutions Preparation

A solution coded as PCL-PANI was prepared by sequentially dispersing 19 mg of (1S)-(+)-10-camphorsulfonic acid (CSA, 99% purity, Sigma Aldrich) and 15 mg of PANI emeraldine base (Mw = 100 kDa, Sigma Aldric) in 3 mL of 1,1,1,3,3,3-Hexafluoroisopropanol-d2 (HFP, 99% purity, Carlo Erba Reagents, Milan, Italy), according to the ratio CSA:PANI, proposed by Qazy et al. [42]. CSA is a small acid conventionally used for doping PANI [43]. The obtained solution was sonicated (Sonics VibraCell VCX 130PB) for 3 min at 20 kHz and left under magnetic stirring for 4 h. Meanwhile, 500 mg of PCL (M_n_ = 80 kDa, Sigma Aldrich) was dispersed in 2 mL of HFP. The blended PCL-PANI solution was obtained by pouring doped PANI into to the viscous PCL solution (25% *wt*/*v*) followed by stirring overnight. Moreover, 500 mg of PCL was also dissolved in 5 mL of HFP obtaining a solution (10% *wt*/*v*) that was used as a control.

### 2.2. Electrospinning Process

The electrospun mats were fabricated through the electrospinning instrument (Linari Engineering S.r.l, Pisa PI, Italy) in horizontal configuration. Briefly, the solutions were loaded into glass syringes with a 21 G needle and then placed on the support of the volumetric pump (Linari Engineering S.r.l), set to deliver a flow rate ranging between 1 and 1.5 mL h^−1^. A constant voltage of 20 kV was applied, obtaining polymeric membranes on the surface of a flat collector placed at 20 cm away from needle. The obtained mats (~100 µm thick) were placed under a ventilated hood overnight to promote the complete evaporation of solvent.

### 2.3. Characterization of Membranes

#### 2.3.1. Field Emission Scanning Electron Microscopy (FESEM)

PCL and PCL-PANI electrospun mats were cut into little piece of 1 cm^2^ and glued on the surface of holders (diameter of 1 cm). The deposition of a thin platinum layer was performed using the sputtering instruments (Quorum Q150s) at 20 mA for 20 s. Topographical features of electrospun membranes were observed through FESEM (Zeiss Supra 40). The diameters of 50 fibers of each image were calculated using Image J software (National Institutes of Health, Bethesda, MD, USA) and reported in terms of the mean ± standard deviation.

#### 2.3.2. Fourier Transform Infrared-Attenuated Total Reflectance Spectroscopy (FTIR-ATR)

The functional groups on the surface of PCL and PCL-PANI membranes, as well as of a film of PANI doped with CSA (PANI-CSA) were detected using the spectroscope Spectrum 100 (Perkin Elmer, Waltham, MA, USA) equipped with the ATR accessory universal ATR (UATR) Thallium Bromoiodide (KRS-5). For each sample, 32 scans were recorded at a wavenumber range between 4000 and 600 cm^−1^, with a resolution of 4 cm^−1^.

#### 2.3.3. In Vitro Stability and Uniaxial Tensile Tests

Three samples for each PCL and PCL-PANI membrane were cut in dog bone-like shapes (Figure S1 in the Supplementary Material). The samples were maintained in phosphate buffer solution (PBS, pH = 7.4) at 37 °C for 1, 3 and 7 d. After each time point, the pH of the PBS solutions was recorded (three measures per sample). The degraded samples were rinsed in MilliQ water and freeze-dried (Scanvac CoolSafe) overnight. The morphological properties of the degraded mats were evaluated using FESEM (in the same conditions reported above) by measuring the diameter of 100 fibers per sample. The hydrolytic degradation of membranes was also quantified, calculating the weight loss percentage (*WL%*) following the equation:

(1) $$WL\%=\;\frac{w_{i\;}-\;w_{f\;}}{w_{i\;}}\;100$$

where *w_i_* and *w_f_* are, respectively, the sample weights before and after the degradation process.

Uniaxial tensile tests were carried out before and after the hydrolytic degradation using MTS QTest™/10 uniaxial tensile apparatus equipped with a 10 N load cell, to investigate the influence of PANI on mechanical behavior, as well as to assess the mechanical stability of electrospun mats. Before each test, the thickness of each sample was measured. Three dog-bone shaped samples for each type of electrospun membrane were used. Each sample was fixed on the grips and tested using a crosshead speed of 2 mm min^−1^. The stress–strain curves were plotted for each sample (n = 3), for calculating Young’s modulus (E), ultimate tensile strength (UTS) and strain at failure (*ε*%) starting from the equation:

(2) $$E\;=\;\frac{\sigma }{\varepsilon }$$

where *σ* and *ε* represent the stress and strain respectively, obtained from:

(3) $$\sigma \;=\;\frac{Load}{Area}$$

(4) $$\varepsilon \;=\;\frac{Extention}{length}$$

The calculated values were reported as mean ± standard deviation.

#### 2.3.4. Cold Atmospheric Plasma Treatment and Water Contact Angle (WCA) Measurement

The cold atmospheric plasma treatment of PCL and PCL-PANI mats was performed using Stylus Plasma Noble (Nadir S.r.l, Veneto, Italy). The samples were positioned on a rigid flat support and covered by a polymeric mesh to avoid the movement of samples generated by the interaction with the argon jet. Cold atmospheric plasma is generated by a high voltage discharge that induces the ionization of argon near the outlet of the gas flow. The treatment was performed by setting the argon flux to 7.5 slm, a high voltage (HV) tension of 10 kV_pp_ and a radiofrequency (RF) power of 9 W. Plasma was generated in the stylus that was then manually moved onto the surface of samples for 30 s.

The wettability of plasma treated and untreated PCL and PCL-PANI samples was measured using the Krüss Drop Shape Analyzer apparatus. A piece (60 mm length × 20 mm width × 0.1 mm thick) of each type of membrane was glued on the surface of a rectangular glass coverslip and a drop of 2 μL was filed on the sample surface through the instrument dispenser. Four WCA measurements for each sample in the initial stage of deposition (t = 0) were recorded and reported (mean ± standard deviation).

Moreover, the time durability of membrane surface wettability was tested by recording the values of WCA at different time points (30 min, 1 h, 2 h, 4 h and 6 h after the plasma treatment). The influence of plasma treatment on nanofiber morphology and hydrolytic degradation was evaluated by analyzing the changes in nanofiber size by measuring the diameters of 100 fibers per sample, using FESEM. The calculated values were reported as mean ± standard deviation.

### 2.4. In Vitro Cell Cultures

HFF-1 fibroblasts (ATCC SCRC-1041, human, foreskin) were cultured in Dulbecco’s Modified Eagle’s medium (DMEM, Gibco, ThermoFisher Scientific, Waltham, MA, USA) supplemented with 15% fetal bovine serum (FBS, Gibco, ThermoFisher Scientific), 2% L-glutamine (Gibco, ThermoFisher Scientific) and 1% penicillin streptomycin (Gibco, ThermoFisher Scientific) and maintained in incubation (37 °C, 5% CO_2_) on tissue culture plates (TCP). Cells at 80–90% confluency were used for reported cell tests. The plasma treated and untreated PCL and PCL-PANI electrospun samples used for cell seeding were prepared by electrospinning the polymeric solution directly on the surface of a round glass coverslip of 12 mm diameter. Prior to cell tests, three samples for each type of electrospun mat were allocated in 24-well plates and sterilized with UV light for 1 h. 2 × 10^4^ cells/well were seeded on electrospun samples as well as on the glass coverslip and plastic (TCP, positive control).

#### 2.4.1. Cell Viability Test

Cell viability was assessed using a metabolic colorimetric assay (Resazurin assay) based on the capacity of viable cells to reduce blue non-fluorescent resazurin dye in pink fluorescent resorufin. Resazurin 1× was obtained by mixing resazurin 10× with complete cell culture medium. After each time point (2, 3 and 7 days), the cell culture medium of each well was replaced with 500 µL of resazurin 1× and incubated 4 h at 37 °C. Thus, 100 µL was placed in black 96-well plates and the fluorescence at 590 nm was detected using the SYNERGY™ HTX multimode plate reader (BioTeK, Winooski, VM, USA). Tests were performed in triplicate.

#### 2.4.2. Cell Microscopy

At 2, 3 and 7 days, samples were rinsed twice in PBS and fixed in formalin-free tissue fixative (Sigma Aldrich, Milan, Italy) for 40 min in a rocking platform at room temperature. The cells were permeabilized with 0.5% Triton X-100 (Sigma Aldrich, Milan, Italy) for 10 min. Sequentially, cells were stained with 1:60 Alexa Fluor 488 Phalloidin (FITC-labelled, BioLegend, San Diego, CA, USA) and 1:100 DAPI (Biolegend, San Diego, CA, USA) to observe the cytoskeleton (F-actin) and nucleus of HFF-1 cells. The stained samples were mounted on rectangular coverslips and imaged with a confocal microscope (Eclipse Ti2 Nikon, Tokyo, Japan).

Cell morphology on electrospun scaffolds was observed through FESEM. The fixed samples were dehydrated by incubating the samples in graded ethanol solutions (EtOH) (30/50/70/80/90/95/100% *v/v* EtOH in water) for 15 min each. The samples were prepared and observed as reported in Section 2.3.1.

### 2.5. Statistical Analysis

Statistical analysis was performed by using a *t*-test, one-way ANOVA and two-way ANOVA for single or multiple comparisons using GraphPad Prism 8.3.0 software (GraphPad Software, San Diego, CA, USA).

## 3. Electrospun Membrane Characterization

### 3.1. Influence of Process Parameters and Solution Composition on Nanofiber Morphology

**Figure 1:**
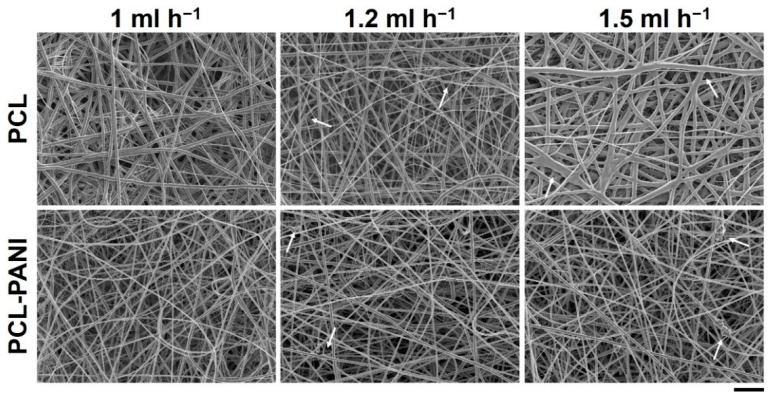
FESEM images of polycaprolactone (PCL) and PCL-polyaniline (PANI) electrospun membranes, obtained by varying the flow rate between 1 and 1.5 mL h^−1^ (scale bar = 10 µm, magnification = 2 K×). Arrows indicate fiber defects.

The effect of the solution flow rate on the nanofiber morphology was evaluated by measuring the diameter of 50 fibers through ImageJ software. Figure 1 shows the FESEM images of PCL and PCL-PANI mats, obtained by setting the solution flow rate to 1, 1.2 and 1.5 mL h^−1^. As shown in Table 1, on enhancing the flow rate, the diameter of the fibers increased from 580 ± 220 to 710 ± 300 nm and from 360 ± 90 to 670 ± 220 nm for the pure PCL and PCL-PANI fibers, respectively. Furthermore, the increase in flow rate resulted in the fabrication of fibers with a heterogeneous morphology, presenting many defects (particularly evident for PCL fibers obtained with a flow rate of 1.5 mL h^−1^).

**Figure 2:**
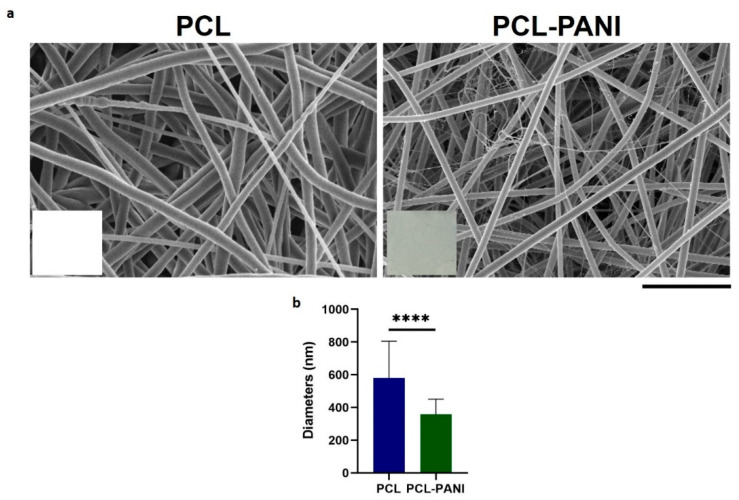
(**a**) FESEM images of optimized PCL and PCL-PANI electrospun membranes (scale bar = 10 µm, magnification = 5 K×). Photograph of PCL and PCL-PANI membranes are reported in the inset; (**b**) Comparison between PCL and PCL-PANI fiber diameters. Statistical difference (**** *p* < 0.0001).

Due to these defects, mats used for further investigation were fabricated with a solution flow rate of 1 mL h^−1^ (Figure 2a). In the inset of FE-SEM images, pictures of electrospun membranes are reported to show the green color of PCL-PANI mats due to the presence of PANI in the solution. PCL membranes presented fibers in a random configuration with some defects (beads). The presence of PANI into the polymeric solution resulted in a significative decrease in the average diameters (Figure 2b). Moreover, PCL-PANI membranes revealed the presence of smooth defect-free nanofibers with an average diameter of 360 ± 90, surrounded by a network of small fibers of 47 ± 14 nm. The addition of PANI into the solution leads to an increase in solution conductivity, as reported in the literature [33,44]. Indeed, the increase in charge density due to PANI enhanced the solution conductivity, causing a decrease in the diameter of electrospun fibers [45] as well as the formation of “spider-like” fibers with smaller diameters [46].

### 3.2. FTIR-ATR Spectroscopy

**Figure 3:**
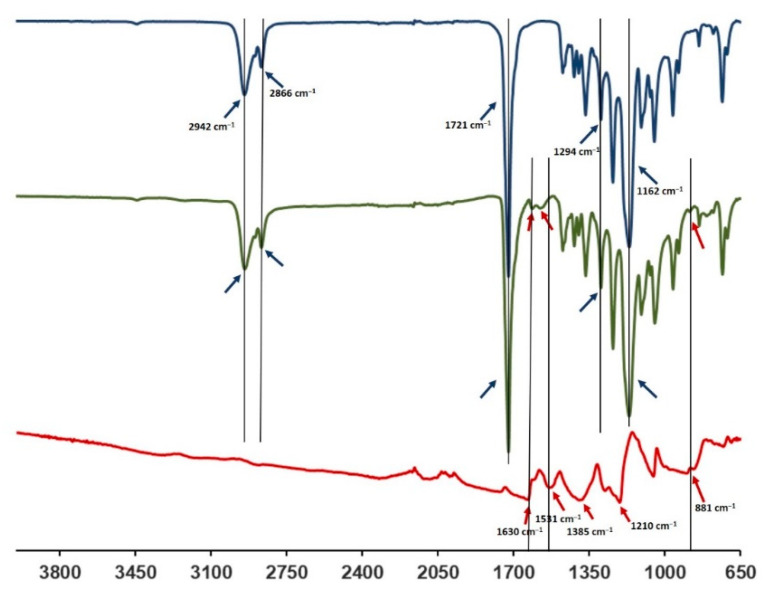
FTIR spectra of PANI-CSA (red), PCL-PANI (green) and PCL (blue).

The FTIR-ATR spectra of the PCL-PANI membrane were compared with those of the PCL membrane and PANI-CSA film (Figure 3) to investigate the surface functional groups of the scaffold, obtained by the bending of materials. The PCL-PANI spectrum showed all the main PCL characteristic peaks at 2942 and 2866 cm^−1^, referring, respectively, to the asymmetric and symmetric stretching of CH_2_ groups [47], at 1721 and 1162 cm^−1^, corresponding to the stretching of C=O [48] and C–O [49], and at 1294 cm^−1^ related to the bands of C–C and C–O of the PCL crystalline phase [50]. Moreover, specific PANI-CSA peaks at 1610, 1562 (stretching vibration of quinoid and benzenoid rings) [51] and 881 cm^−1^ (N–H bending of amine peak) [35] were also detected on the surface of PANI electrospun membranes.

### 3.3. Evaluation of Membranes Stability and Mechanical Properties

**Figure 4:**
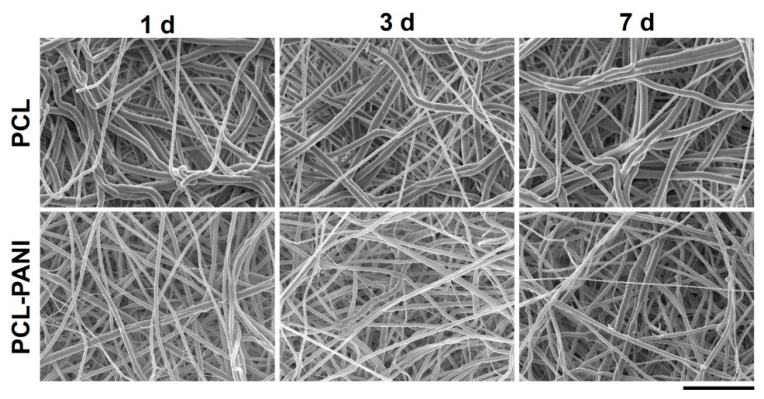
FESEM images of PCL and PCL-PANI mats after 1, 3 and 7 days of hydrolytic degradation (scale bar = 10 µm, magnification = 5 K×).

In vitro degradation tests were performed to evaluate the behavior of electrospun nanofibers after incubation in PBS solution (T = 37 °C and pH = 7.4) for 1 week, mimicking physiological conditions. Figure 4 and Table S1 demonstrate the absence of significant changes in the morphology of PCL-PANI and PCL nanofibrous scaffolds after incubation. However, as shown in Figure S2 (supplementary material), the weight loss of PCL-PANI mats during the incubation period was notably higher (*p* < 0.05) than that recorded for PCL mats (5.7 ± 2.1% and 1.11 ± 0.5 for PANI and PCL mats after 7 days in PBS, respectively). As mentioned above, the addition of PANI induced a reduction in the size of blended fibers. According to a previous work [52], smaller nanofiber diameters might correlate with an increase in weight loss. Moreover, following the degradation of the PCL-PANI membranes, the increase in membrane weight loss corresponded to a slight decrease in the pH of the PBS medium (Figure S3), reaching pH 7.25 after 7 days.

**Figure 5:**
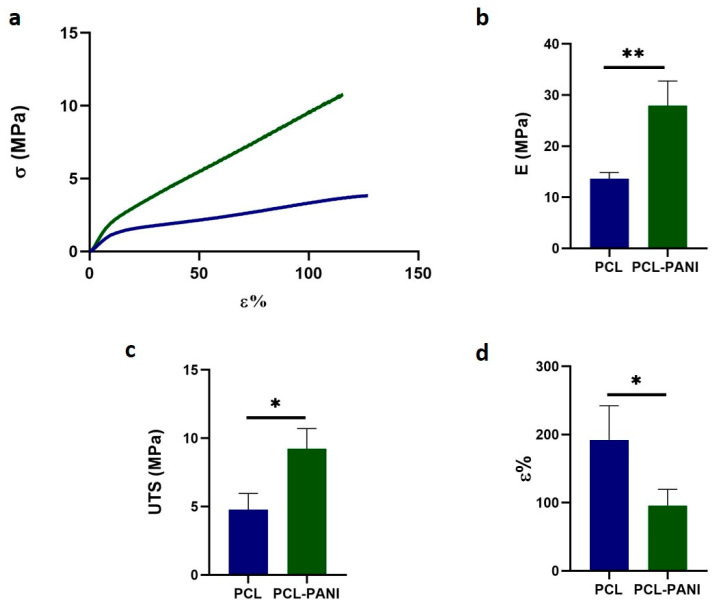
Mechanical properties of PCL and PCL-PANI electrospun mats: (**a**) stress-strain curves; (**b**) Young’s modulus (E); (**c**) ultimate tensile strength (UTS) and (**d**) strain at failure (*ε*). Statistical difference (* *p* < 0.05; ** *p* < 0.005).

Table 2 shows the mechanical properties of mats, determined from the stress– strain curves of PCL and PCL-PANI electrospun samples (Figure 5a). The Young modulus (E), ultimate tensile strength (UTS) and elongation at break (*ε*%) of the PCL fibers were calculated to be 13.6 ± 1.2 MPa, 4.8 ± 1.2 MPa and 192.2 ± 50.2%. The E and UTS significantly increased for PCL-PANI membranes, respectively, to 27.9 ± 4.8 and 9.2 ± 1.5 MPa (Figure 5b,c). However, a relevant decrease in *ε*% to 95.8 ± 23.7% was observed in the blended scaffolds (Figure 5d). These results demonstrate that the presence of brittle PANI led to the increase in stiffness of the electrospun membranes [53].

Moreover, the mechanical stability of membranes incubated in PBS solution at 37 °C was also measured. After 1 week of incubation of pure PCL and PCL-PANI mats, no significant change in the mechanical properties of electrospun membranes was detected (Figure S4).

### 3.4. Atmospheric Plasma Treatment of Electrospun Membranes

Atmospheric plasma treatment should result in the modification of the surface properties of treated materials. For this reason, the morphology, water contact angle and in vitro biodegradation of membranes were assessed after plasma treatment.

**Figure 6:**
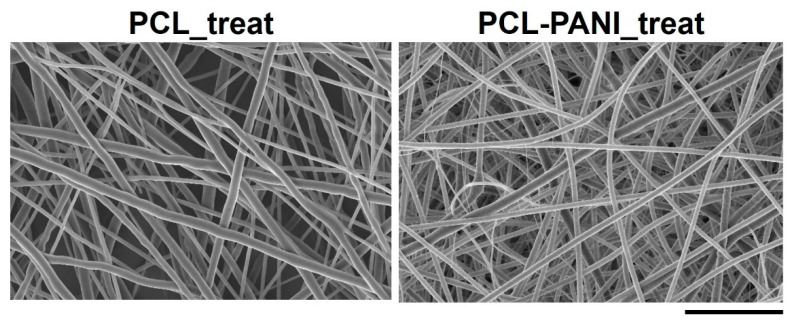
FESEM micrograph of PCL and PCL-PANI after Ar cold atmospheric plasma treatment (scale bar = 10 µm, magnification = 5 K×). No alterations in fiber morphology were detected after plasma treatment compared to untreated samples.

Figure 6 shows the FESEM images of Ar treated PCL and PCL-PANI mats (coded as PCL_treat and PCL-PANI_treat). After plasma treatment, no significant morphological change was observed, confirming that the plasma treatment did not affect the bulk properties of the nanofibers.

**Figure 7:**
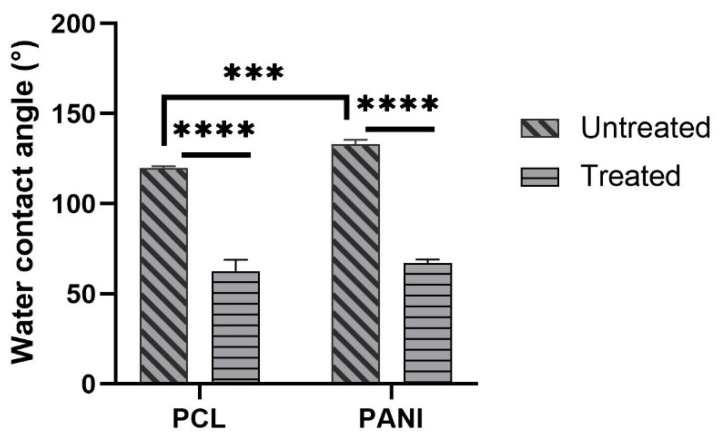
Water contact angle measures of PCL and PANI electrospun membranes before and after plasma treatment. Statistical difference (*** *p* < 0.001; **** *p* < 0.0001).

The measure of contact angle of membranes was performed to analyze the effect of PANI, as well as plasma treatment on the surface behavior of blended mats. The values of contact angles for PCL and PCL-PANI mats are reported in Figure 7. As the histogram shows, the presence of PANI in the membrane results in an increase in the water contact angle of the mats from 119° ± 1° to 133.5° ± 2.2° (respectively, for PCL and PCL-PANI nanofibers).

The decrease in the water contact angle for treated PCL and PCL-PANI to 62.7° ± 6.3° and 67.1° ± 2° demonstrated the effective modification of the surface after plasma treatment.

The time durability after atmospheric plasma treatment of electrospun mats was assessed by monitoring the WCAs of PCL_treat and PCL-PANI_treat samples up to 6 h. As shown in Table 3, no significant changes in WCA values were detected, demonstrating that the plasma treatment lasts as long as cells are seeded on the membranes.

Since an improvement in surface wettability leads to a greater interaction between water and the membrane, in vitro biodegradation of PCL_treated and PCL-PANI_treated was assessed through FESEM by measuring the diameter of 100 fibers per sample. As shown in Figure S5, the stability of treated mats in the physiological environment (PBS solution at 37 °C) was confirmed up to 7 days. Moreover, no significative variation in the PCL_treat and PCL-PANI_treat sample diameters (Table S2) was recorded.

### 3.5. Evaluation of Cells Viability and Attachment

**Figure 8:**
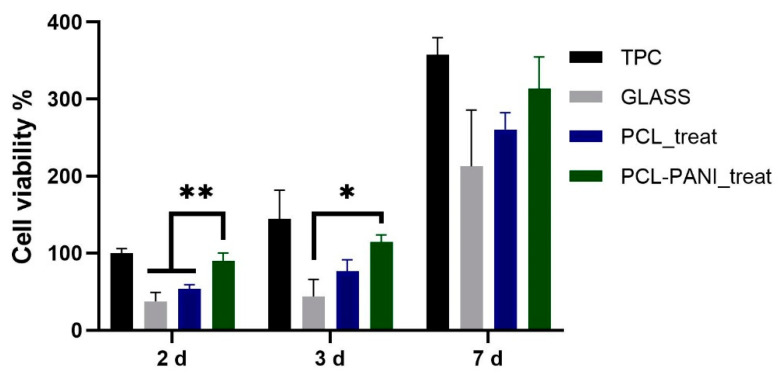
Evaluation of HFF-1 viability on different substrates after 2, 3 and 7 days of culture. Statistical difference (* *p* < 0.05; ** *p* < 0.005).

The influence of PANI on the viability of HFF-1 fibroblasts was assessed with the resazurin assay. The viability of cells on TCP at 2 days was used as a control. As shown in Figure 8, the metabolic activity of the seeded cells on all the substrates was significantly increased during the culture period. A higher level of viable cells was detected for PCL-PANI_treat on day 2 and 3 compared to glass coverslip (GLASS)and PCL_treat, indicating that the blended scaffold better promotes attachment and growth of the cells. Moreover, cell viability for seeding at 7 days, was comparable for all the scaffolds, as well as for the control.

**Figure 9:**
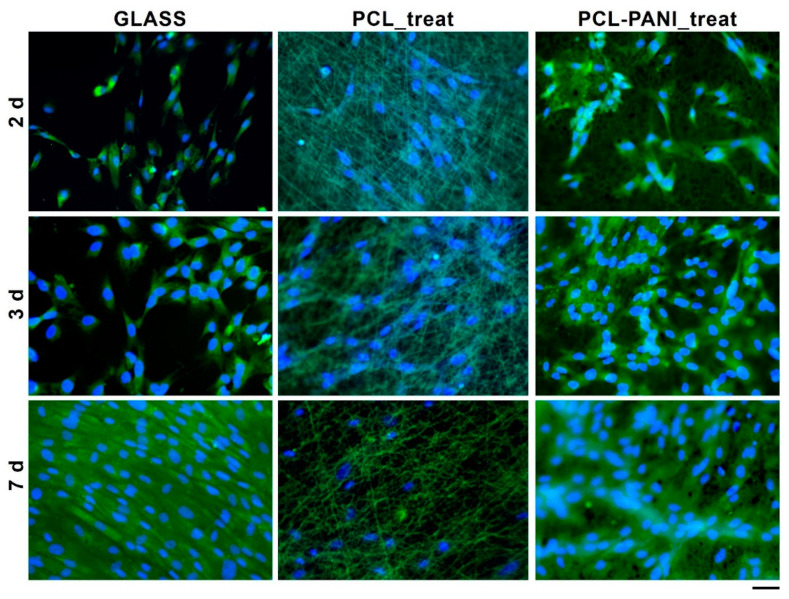
Fluorescent microscopy images of HFF-1 stained with FITC phalloidin (actin filaments) and DAPI (nuclei) after 2, 3 and 7 days of culture (scale bar = 50 µm, magnification = 20×).

The morphology of HFF-1 fibroblasts on different culture substrates was assessed by staining cell nuclei and actin filaments with DAPI (blue) and phalloidin (green), respectively (Figure 9).

**Figure 10:**
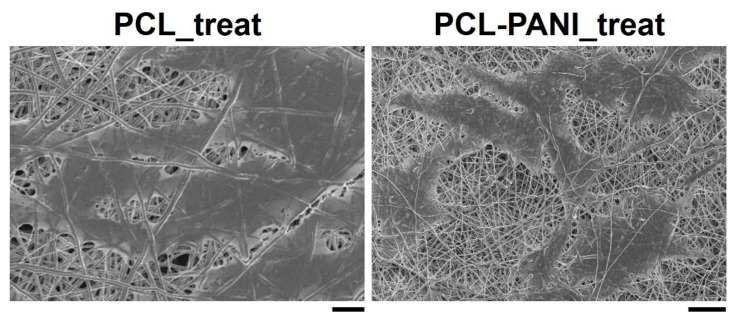
FESEM micrograph of HFF-1 (scale bar = 10 µm, magnification = 1 K×) on PCL_treat and PCL-PANI_treat samples (scale bar = 20 µm, magnification = 1 K×) after 7 days of culture.

According to the results obtained from the resazurin assay, the number of cells on the PCL-PANI_treat mats was higher than that on the PCL_treat and GLASS samples at days 2 and 3. Therefore, after 7 days, the cells were uniformly distributed along the direction of the electrospun fibers. The attachment of HFF-1 cells on the electrospun mats was also investigated through FESEM microscopy (Figure 10). The typical spindle-like shape of the fibroblasts was observed for PCL-PANI_treat samples, where the cells appeared healthier and better attached, compared to cells growth on PCL_treat mats.

## 4. Discussion

The use of CPs for fabricating “smart” scaffolds for TE has been extensively investigated in recent years thanks to their capability to improve the adhesion, viability, and morphology of cells, promoting the regenerative process of several tissues. In this context, PANI is one of the most investigated materials, thanks to its interesting properties. In this work, we propose the blending of PCL and PANI for the fabrication of conductive electrospun nanofibrous membranes. Firstly, an investigation of the influence of electrospinning parameters on the morphological properties of nanofibers was performed. The solution flow rate was determined to be the leading parameter that affects the morphological behavior of fibers, due to its influence on the shape of the generated drops, the direction of the polymeric jet, the formation and shape of the Taylor cone, and the fiber collection zone [54]. The diameters of PCL and PCL-PANI nanofibers (Figure 1) and beads increased with the increase in flow rate from 1 to 1.5 mL h^−1^. It is known that a high flow rate of a solution causes the ejection of a large amount of solution from the needle correlated with the formation of large defects. Furthermore, in travelling between the needle and the collector, the solvent was unable to completely evaporate, implying the fusion of fibers and an increase in their diameters [54,55]. The topographic behavior influenced the interaction between the scaffold and cells. In particular, small fibers could better regulate the cell response on electrospun membranes [56]. These results led to set the flow rate to 1 mL h^−1^ allowing us to obtain defect-free and smaller diameter fibers. The morphology of PCL-PANI mats was characterized and compared to PCL mats (Figure 2). The presence of PANI increased the solution conductivity and the charge density [57,58], causing two main changes in morphology of fibers: (i) the reduction in the average diameter of the fibers, caused by the electrical force that elongates the jet [33]; (ii) the formation of much thinner “spider-like” fibers, generated by the secondary jets [46]. FTIR spectra (Figure 3) demonstrated the incorporation of PANI, by displaying both characteristic groups of PCL and PANI-CSA on the PCL-PANI mats. The specific peaks of PANI-CSA, related to the stretching vibration of quinoid and benzenoid rings, were slightly shifted (respectively, from 1531 to 1562 cm^−1^ and from 1630 and 1610 cm^−1^) in the PCL-PANI mats due to the interaction between PCL and PANI-CSA [59]. The other characteristic peaks of PANI-CSA at 1385 and 1210 cm^−1^, attributed to C=N^+^ stretching and N-H bending, respectively, were not detected for PANI electrospun mats [51]. The hydrolytic degradation tests demonstrated the stability of electrospun mats in the physiological environment (Figure 4). Indeed, no significant changes in the average diameter of fibers after hydrolytic degradation were recorded, as shown in Table S1. However, a higher weight loss of PCL-PANI mats during incubation (Figure S2) was detected. Some studies investigated the correlation between scaffold morphology and weight loss, reporting that the reduction in nanofiber diameter resulted in a more evident weight loss due to the increase in the ratio of surface area to volume of scaffolds [52,60]. Taking into account these considerations, the increase in weight loss of blended membranes could be determined by the decrease in diameters of the PCL-PANI nanofibers. Moreover, the increase in weight loss could be correlated to the decrease in pH values of the PBS solution during hydrolytic degradation [61] (Figure S3), attributed to the slight quantities of acid leaching from the surface of blended membranes, which has already been observed in the literature [62]. Scaffold mechanical properties influence the behavior of the cell, such as cell attachment, proliferation and tissue growth [36]. In the presence of PANI, a change in the mechanical properties of electrospun mats was observed (Figure 5). PCL is a thermoplastic polymer with a slow degradation rate and high flexibility [63]. On testing the mechanical properties of scaffolds, it was concluded that the presence of the brittle PANI in the PCL-PANI blend nanofibers produced less elastic and stronger mats [33,64]. The mechanical properties were maintained even after soaking samples in physiological condition for 1 week. No significant changes in measured values were detected (Figure S4). This result demonstrated that the mechanical stability of PCL-PANI mats was ascribed to PCL. Previous work showed that the reduction in mechanical properties of PCL-based nanofibers becomes significant after 2 months of hydrolytic degradation [65]. The surface wettability is one of the most important behaviors that affects the interactions between scaffolds and cells, and consequently, cell adhesion and viability. For this reason, in this work, we propose the surface modification of PCL-PANI nanofibers with cold atmospheric Ar plasma. Plasma treatment only modifies the scaffold surface without altering its morphology. For this reason, the changes in morphology and degradation of the nanofibers after plasma treatment were assessed. According to Maffei et al. [40], the cold atmospheric plasma treatment did not affect the morphology (Figure 6) or the degradation rate (Figure S5, Table S2) of the electrospun mats. The wettability of nanofibers was assessed by measuring the water contact angle of the mats. A decrease in the hydrophilicity of the scaffold was detected as result of the incorporation of PANI (Figure 7). Similar results were obtained by Farkhondehnia et al. [66] for composite PLGA/PCL/PANI nanofibrous scaffolds. Many studies reported that the hydrophobic properties of the surfaces (WCA > 90°) interfere with cell adhesion and attachment on the scaffold [67,68]. On the other hand, when the WCA is between 60° and 80°, higher levels of cell attachment are obtained [69]. The WCA of the PCL and PCL-PANI mats decreased following surface modification with Ar plasma (Figure 7), maintaining hydrophilic properties up to 6 h (Table 3). According to previous studies, the decrease in the contact angle of the PCL based mats after Ar plasma treatment can be related to the increase in the substrate surface energy and to the creation of binding sites, as well as the attachment of polar groups [70,71]. The biocompatibility of PANI-blended electrospun fibers has previously been reported by other groups. Farkhondehnia et al. [66] investigated the cytotoxicity of PLGA/PCL/PANI mats. They showed that A-172 nerve cells attached and proliferated on the blended fibers. In another piece of work by Chen et al. [33], it was proposed that the fabrication of aligned PCL-PANI nanofibers demonstrated that the increase in PANI content in the fibers did not affect the viability of C2C12 myoblasts. Here, the biocompatibility of scaffolds was assessed by culturing HFF-1 fibroblasts on plasma untreated and treated electrospun mats. The hydrophobicity of PCL and PCL-PANI mats hindered cell attachment to the scaffolds, resulting in a low viability at 2 days of culture. Conversely, the results obtained from the viability assay on treated mats demonstrated the biocompatibility of blended fibers (Figure 8), showing that the presence of PANI did not exert any toxic effect on cells. After 2 and 3 days of culture, a greater cell viability was detected on PCL-PANI_treat samples. As previously reported in the literature, the inherent electroactivity of PANI promoted the proliferation of cells cultured on PANI-based scaffolds [62,72,73,74]. Moreover, this result could be explained considering that the smaller diameters of PCL-PANI_treat fibers better mimic the natural ECM than the larger PCL_treat fibers and plane surface of the GLASS [64], thus, promoting cell attachment and growth. No significant difference in cell viability was observed at 7 days when cells reached the confluence state. The morphological behavior of cells was investigated through DAPI/Phalloidin staining. The nuclei and cytoskeletons of cells were detected on the substrates. The cells cultured on PCL-PANI_treat showed a more elongated morphology than those on PCL_treat mats. Interestingly, as is evident in Figure 9, the PCL_treat fibers were more visible than PCL-PANI_treat fibers, due to autofluorescence of PCL [75]. According to a previous work by Li et al. [64], this phenomenon was slightly attenuated by the presence of PANI in the blended nanofibers due to the non-autofluorescence of PANI, as well as the smaller dimension of PCL-PANI_treat nanofibers. The FESEM images (Figure 10) of cells grown on mats confirmed the higher cell attachment on PCL-PANI_treat substrates.

## 5. Conclusions

In this work, the preparation and characterization of conductive membranes for tissue engineering applications was performed. The electrospinning technique was selected for fabricating fibrous PCL-PANI blended membranes which exhibited fibers with smaller diameters compered to PCL fibers. The presence of PANI was confirmed through FTIR-ATR spectroscopy, showing the main characteristic peaks of the doped form of the conductive material (PANI-CSA). Moreover, the addition of brittle PANI resulted in an increase in the mechanical stiffness of the PCL-PANI electrospun membranes. The WCA of PCL-PANI membranes confirmed the hydrophobic properties of PCL-PANI, which can hinder cell attachment and viability. Therefore, the electrospun mats were treated with atmospheric Ar plasma, resulting in a reduction in the WCA being obtained; from 133.5 ± 2.2° for untreated samples, to 67.1 ± 2° for treated samples. The in vitro stability of PCL-PANI and PCL-PANI_treated membranes was demonstrated by incubating samples in PBS solution for 7 days. The attachment and viability of HFF-1 cells were enhanced when compared to PCL alone, thanks to the combination of topographic and electroactive properties that the presence of PANI confers to electrospun scaffolds. This confirmed that the fabricated scaffolds represent promising substrates for tissue engineering applications.

## Figures and Tables

**Table 1:** Correlation between flow rate used for PCL and PCL-PANI membrane preparation and diameter of fibers.

	Flow Rate (mL h^−1^)	Diameter (nm)
**PCL**	1	580 ± 220
	1.2	670 ± 210
	1.5	716 ± 300
**PCL-PANI**	1	360 ± 90
	1.2	640 ± 180
	1.5	670 ± 220

**Table 2:** Mechanical properties of PCL and PCL-PANI mats: Young’s modulus (E), ultimate tensile strength (UTS), strain at failure (*ε*).

	E (MPa)	UTS (MPa)	*ε* (%)
**PCL**	13.6 ± 1.2	4.8 ± 1.2	192.2 ± 50.2
**PCL-PANI**	27.9 ± 4.8	9.2 ± 1.5	95.8 ± 23.7

**Table 3:** Time durability after atmospheric Ar plasma treatment of PCL_treat and PCL-PANI_treat.

*Time*	Water Contact Angle (Mean ± Standard Deviation °)	
	PCL_Treat	PCL-PANI_Treat
**30 min**	64.1 ± 4.1	65.6 ± 3.6
**1 h**	63.5 ± 5.3	67.3 ± 2.9
**2 h**	63 ± 6.7	67 ± 2.2
**4 h**	62.7 ± 6.8	66.2 ± 4.6
**6 h**	63.4 ± 2.6	66.8 ± 2.5

## Data Availability

The data presented in this study are available on request from the corresponding author.

## Supplementary Materials

The following are available online at https://www.mdpi.com/2306-5354/8/2/24/s1, Figure S1: Dog-bone shape sample of PCL-PANI electrospun membranes, Figure S2: Evaluation of PCL and PCL-PANI mats weight loss % (WL %) after 1, 3 and 7 days of hydrolytic degradation, Figure S3: Evaluation of pH of PBS solution after 1, 3 and 7 days of hydrolytic degradation of PCL and PCL-PANI. Table S1: Evaluation of PCL and PCL-PANI average diameter after 1, 3 and 7 days of hydrolytic degradation by measuring the length of 100 fibres through ImageJ software, Figure S4: Evaluation of PCL and PCL-PANI mechanical stability, Figure S5: FESEM images of PCL_treat and PCL-PANI_treat mats after 1, 3 and 7 days of hydrolytic degradation, Table S2: Evaluation of PCL_treat and PCL-PANI_treat average diameter after 0, 1, 3 and 7 days of hydrolytic degradation by measuring the length of 100 fibres through ImageJ software.
