# Supplementary material for: Biocompatible Electrospun Polycaprolactone-Polyaniline Scaffold Treated with Atmospheric Plasma to Improve Hydrophilicity
Source: Bioengineering (Basel). 2021 Feb 13;8(2):24. doi: 10.3390/bioengineering8020024 (PMC7918921; doi:10.3390/bioengineering8020024)
Supplement: Supplementary file 1 [file bioengineering-08-00024-s001.pdf]

bioengineering

Supplementary material

Biocompatible Electrospun Polycaprolactone-Polyaniline Scaffold Treated with Atmospheric Plasma to Improve Hydrophilicity

Michela Licciardello [1,2,3], Gianluca Ciardelli [1,2,3,4] and Chiara Tonda-Turo [1,2,3,*]

1 Department of Mechanical and Aerospace Engineering, Politecnico di Torino, 10129 Turin, Italy; michela.licciardello@polito.it (M.L.); gianluca.ciardelli@polito.it (G.C.)
2 Polito BIOMedLAB, Politecnico di Torino, 10129 Turin, Italy
3 Interuniversity Center for the promotion of the 3Rs principles in teaching and research, 56122 Pisa, Italy
4 CNR-IPCF, National Research Council-Institute for Chemical and Physical Processes, 56124 Pisa, Italy
* Correspondence: chiara.tondaturo@polito.it

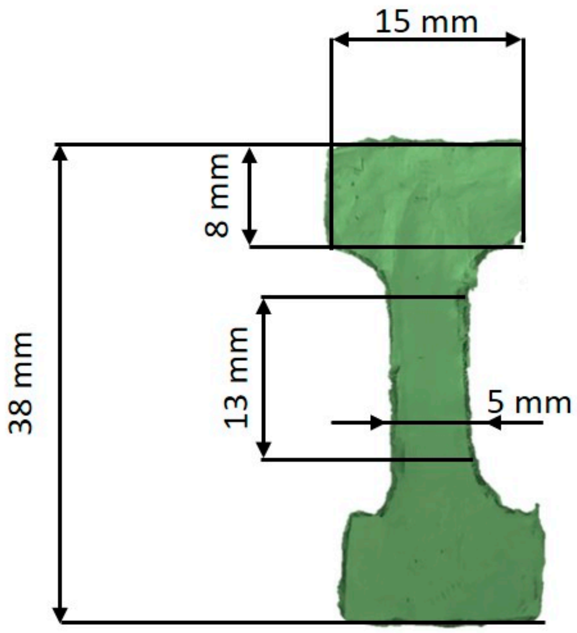

Figure S1. Dog-bone shape sample of PCL-PANI electrospun membranes.

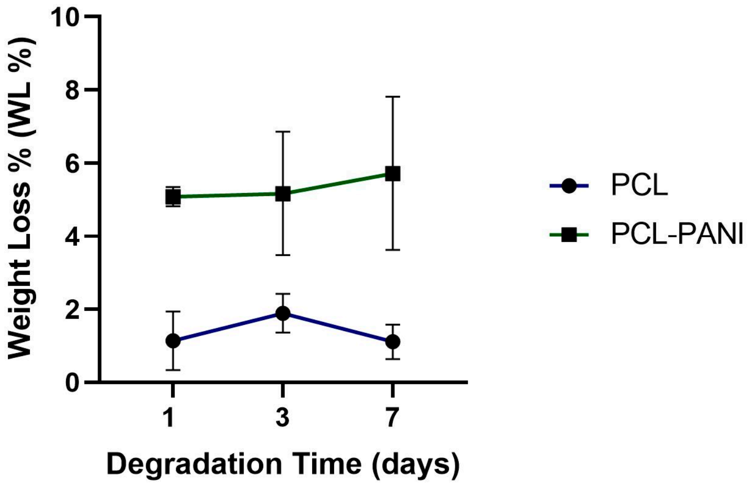

Figure S2. Evaluation of PCL and PCL-PANI mats weight loss % (WL %) after 1, 3 and 7 days of hydrolytic degradation.

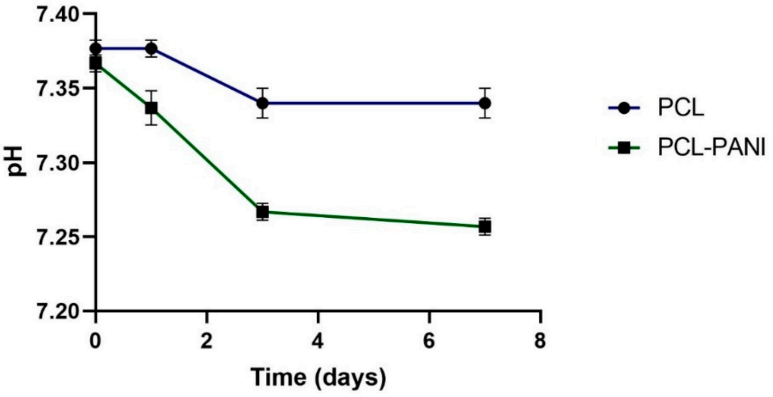

Figure S3. Evaluation of pH of PBS solution after 1, 3 and 7 days of hydrolytic degradation of PCL and PCL-PANI.

Table S1. Evaluation of PCL and PCL-PANI average diameter after 1, 3 and 7 days of hydrolytic degradation by measuring the length of 100 fibres through ImageJ software.

Time	*Diameter (mean ± standard deviation nm)*	
	**PCL_treat**	**PCL-PANI_treat**
**1 d**	512 ± 175	332 ± 123
**3 d**	559 ± 203	325 ± 102
**7 d**	520 ± 80	331 ± 133

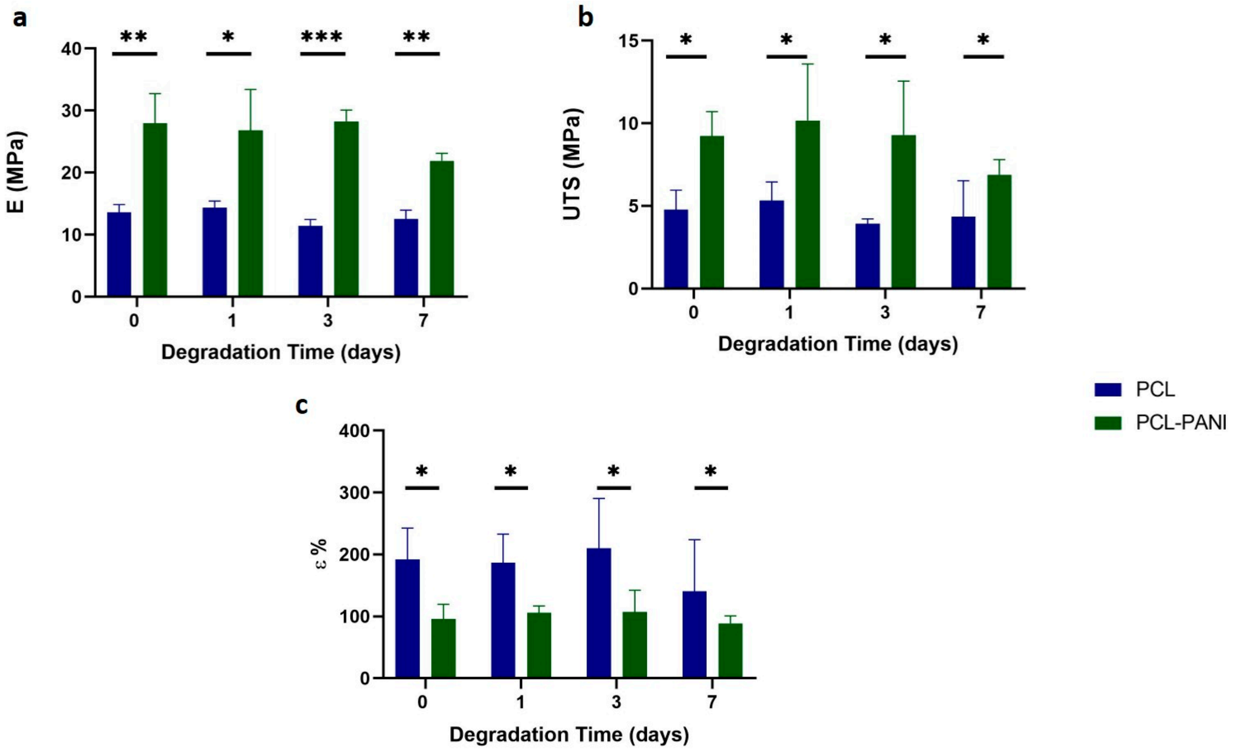

Figure S4. Evaluation of PCL and PCL-PANI mechanical stability a) Young's modulus (E); b) ultimate tensile strength (UTS) and c) strain at failure (ε) after 1, 3 and 7 days of hydrolytic degradation. Statistical difference (*$p < 0.05$; **$p < 0.005$; ***$p < 0.0005$).

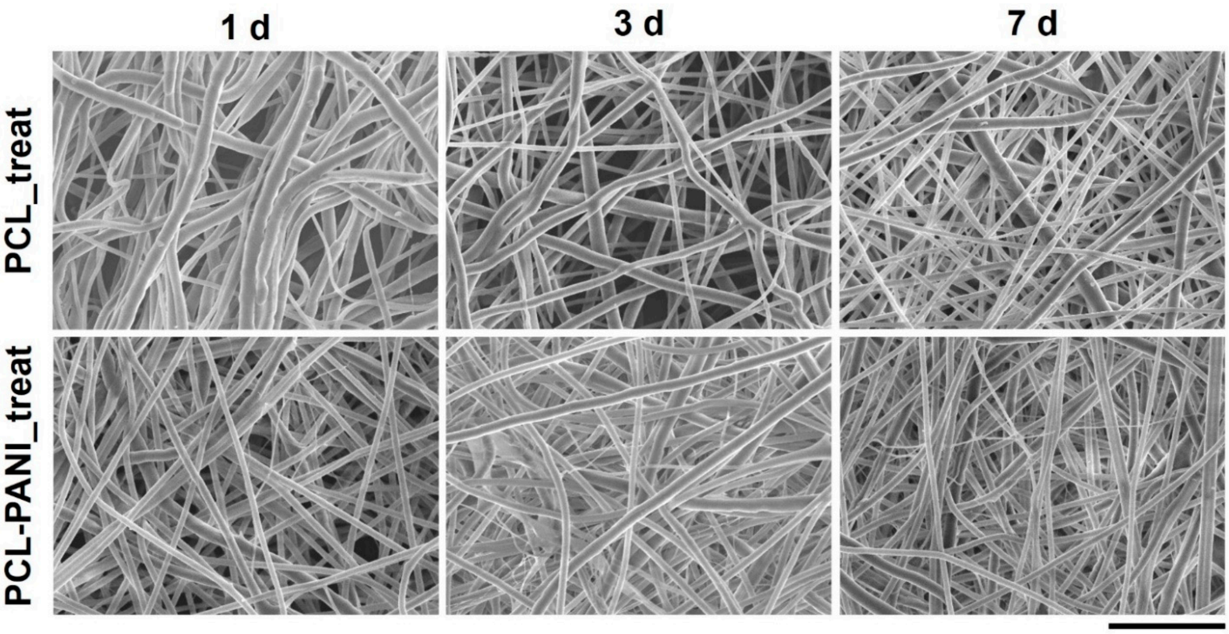

Figure S5. FESEM images of PCL_treat and PCL-PANI_treat mats after 1, 3 and 7 days of hydrolytic degradation (scale bar = 10 µm, magnification = 5 K X).

Table S2. Evaluation of PCL_treat and PCL-PANI_treat average diameter after 0, 1, 3 and 7 days of hydrolytic degradation by measuring the length of 100 fibres through ImageJ software.

Time	*Diameter (mean ± standard deviation nm)*	
	**PCL_treat**	**PCL-PANI_treat**
**0 d**	571 ± 235	358 ± 83
**1 d**	534 ± 22	343 ± 105
**3 d**	516 ± 172	356 ± 98
**7 d**	515 ± 197	367 ± 105
